# Investigation of the Correlation Between Aspects of Sexuality, Self-Esteem, Anxiety, and Depression in Health Professionals in Central Greece

**DOI:** 10.7759/cureus.90795

**Published:** 2025-08-23

**Authors:** Krystallia Gkouletsa, Lampros Mitrakas, Evangelos C Fradelos, Vissarion I Bakalis, Stella Zetta, Maria Saridi

**Affiliations:** 1 Urology, University Hospital of Larissa, University of Thessaly, Larissa, GRC; 2 Nursing, University of Thessaly, Larissa, GRC

**Keywords:** anxiety, depression, health personnel, self-esteem, sexuality

## Abstract

Background: Sexuality is a multidimensional aspect of human functioning that is closely linked to mental health. Among healthcare professionals, high emotional demands, irregular schedules, and burnout can disrupt both psychological balance and sexual well-being. However, research exploring this intersection in clinical populations remains limited, especially in Greece. This study aims to examine the associations between aspects of sexuality, self-esteem, anxiety, and depression, as well as demographic and occupational differences. Methodology: A cross-sectional survey was conducted among 202 healthcare professionals working in primary, secondary, and tertiary care units in Central Greece. Participants included physicians, nurses, and allied health professionals, such as pharmacists. Five validated psychometric instruments were used: the Sexuality Scale, the Brief Sexual Attitudes Scale, the Satisfaction With Sex Life Scale, the Rosenberg Self-Esteem Scale, and the Depression Anxiety Stress Scales-21 (DASS-21). Statistical analyses included descriptive statistics, Pearson correlations, t-tests, ANOVA, and multiple linear regression. Age groups were categorized as 20 to 29, 30 to 39, and ≥40 years. Educational level was classified as undergraduate (basic university degree) or postgraduate (MSc/PhD). Results: Participants reported moderate levels of sexual confidence (mean (M)=8.52), low sexual preoccupation (M=-1.95), and moderate sexual satisfaction (M=4.50). Sexuality was positively correlated with self-esteem (r=0.35, p<0.01) and negatively correlated with anxiety (r= -0.30, p<0.01) and depression (r= -0.28, p<0.01). Women reported lower sexual confidence and self-esteem and higher sexual preoccupation compared to men. Physicians scored higher on sexuality and self-esteem than nurses and pharmacists. Postgraduate participants showed higher means across psychological and sexual indicators. No significant differences were observed by age group. Conclusions: This study underscores the strong interplay between self-esteem and psychological symptoms in relation to sexuality among healthcare professionals. Self-esteem emerged as the most robust positive predictor of sexuality, while anxiety and depression were significant negative predictors. Notable differences were also observed based on gender, profession, and education level. These findings suggest the need for integrated mental health and sexual wellness initiatives tailored to clinical staff. Importantly, this is the first known study to explore these relationships among healthcare professionals in Central Greece, providing a valuable foundation for future research and intervention planning.

## Introduction

Sexuality is a fundamental aspect of human identity and functioning, significantly influencing emotional connection, life satisfaction, and psychological stability. It encompasses biological, psychological, and social dimensions, all of which are influenced by individual and contextual variables. Thus, sexuality is strongly associated with quality of life [1]. For health professionals, maintaining healthy sexual and psychological functioning is particularly crucial, as these individuals are frequently exposed to emotionally intense situations, irregular working hours, and burnout-related stressors [2]. In this study, the term 'sexuality' refers specifically to measurable psychosocial dimensions such as sexual self-esteem, sexual preoccupation, sexual assertiveness, and subjective satisfaction with sex life. These components are assessed through standardized tools, including the Sexuality Scale by Snell & Papini and the Satisfaction with Sex Life Scale [3,4]. The broader conceptual domains of sexual orientation, identity, or behavior were not within the scope of our investigation. Although the literature has progressively addressed mental health among healthcare workers, sexuality remains an underexplored topic. Most relevant studies focus on patients or the general population, neglecting the needs and experiences of clinicians themselves. Health professionals are uniquely positioned; they are both caregivers and individuals affected by the same emotional and relational dynamics they are trained to manage in others [5]. Recent meta-analyses confirm that stress, anxiety, and emotional exhaustion during clinical work are associated with impaired emotional intimacy and decreased sexual satisfaction [6]. Even minor changes in one’s sexual life, resulting in either dissatisfaction or satisfaction, can significantly affect psychological well-being [7]. Research indicates that sexual dissatisfaction is closely linked to symptoms of anxiety, depression, and low self-esteem [8,9]. Self-esteem, in turn, has a well-established connection with both general well-being and sexual satisfaction [10,11]. It influences how individuals perceive their body and their attractiveness as well as their capacity to engage in and enjoy intimate relationships [10]. Low self-esteem may hinder open communication with partners and increase vulnerability to psychological distress, which may further impact sexual function [12].

Moreover, sociocultural factors, including gender norms and educational levels, can shape experiences of sexuality and self-perception [13]. The COVID-19 pandemic amplified these dynamics, with several studies documenting decreased sexual desire and function, especially among female healthcare workers and those experiencing heightened anxiety or stress [14]. Despite these findings, comprehensive studies that investigate the interplay between sexuality, psychological symptoms, and self-esteem in health professionals are still scarce. This study aims to fill that gap by examining correlations between sexuality, self-esteem, anxiety, and depression in a sample of healthcare professionals in Central Greece.

## Materials and methods

A cross-sectional quantitative study was designed to explore psychological and sexual health in health professionals. We applied the method of convenience sampling, whereby eligible participants were recruited through professional networks, institutional email invitations, and on-site distribution of printed questionnaires at healthcare facilities. Participation was voluntary, and no financial or other incentives were offered. The sample participants were employed in diverse healthcare settings of primary, secondary, and tertiary care, including hospitals, clinics, and primary care units in Central Greece. Eligible participants were licensed healthcare workers, including physicians, nurses, and pharmacists (the only allied health professionals represented in the sample), who voluntarily consented to participate. Age groups were categorized as 20 to 29, 30 to 39, and ≥40 years. Educational level was classified as undergraduate (basic university degree) or postgraduate (MSc/PhD). Inclusion criteria included current employment in a healthcare setting, fluency in Greek, and willingness to complete self-administered questionnaires. Ethical approval for the study was obtained from the internal Ethics Committee of the Department of Nursing of the University of Thessaly (approval no. 1677, 27-12-2023), and all procedures conformed to the Helsinki Declaration for human research. We utilized five standardized instruments for data collection.

Sexuality Scale by Snell & Papini

This scale comprises three subscales measuring sexual self-esteem (10 items), sexual preoccupation (six items), and sexual assertiveness (six items). Responses are recorded on a 5-point Likert scale ranging from 1 (“not at all characteristic of me”) to 5 (“very characteristic of me”). Scores are computed separately for each subscale, with higher scores reflecting higher self-esteem, greater preoccupation, or stronger assertiveness in sexual contexts [3]. Taylor and Francis grant permission to use this scale for academic purposes, subject to specific limitations, which are fulfilled in this work.

Brief Sexual Attitudes Scale (BSAS)

This scale evaluates individual attitudes across four domains, namely permissiveness (10 items), birth control (three items), communion (five items), and instrumentality (five items). Items are rated on a 5-point Likert scale. Subscale scores are calculated by averaging item responses, where higher values indicate stronger endorsement of the respective sexual attitude [15]. Taylor and Francis grant permission to use this scale for academic purposes, subject to specific limitations, which are fulfilled in this work.

Satisfaction with Sex Life Scale (SWSLS)

This scale includes a single item assessing overall satisfaction with one’s sex life. Respondents rate their satisfaction on a 7-point Likert scale, with endpoints ranging from “completely dissatisfied” to “completely satisfied.” Higher scores reflect greater subjective satisfaction [4]. This scale has been used in numerous peer-reviewed studies without explicit permission or licensing requirements. It is considered to fall under the category of publicly available, free-access single-item instruments that do not require author or publisher authorization for academic and non-commercial research use.

Rosenberg Self-Esteem Scale (RSES)

This is a widely used 10-item instrument measuring global self-worth. Each item is rated on a 4-point scale from “strongly agree” to “strongly disagree.” Total scores range from 0 to 30, with higher scores indicating greater self-esteem. The RSES demonstrates strong internal consistency and construct validity across diverse populations [16]. The scale is in the public domain, meaning it can be used without charge and without notifying the Sociology Department of the University of Maryland, United States of America.

Depression Anxiety Stress Scales-21 (DASS-21)

This is a 21-item instrument comprising three subscales: depression, anxiety, and stress (seven items each). Participants rate the frequency or severity of each item over the past week on a 4-point scale ranging from 0 (“did not apply to me at all”) to 3 (“applied to me very much or most of the time”). Each subscale score is calculated by summing the relevant items and multiplying the result by two, producing a final score ranging from 0 to 42 per domain [17,18]. The questionnaire is in the public domain, and so permission is not required to use it. The DASS questionnaires and scoring key may be downloaded from the DASS website and copied without restriction. Participants completed the questionnaires anonymously, either in printed or electronic format. There was no time limit for their answer. Researchers ensured data confidentiality and anonymity throughout the process. Data were analyzed using SPSS Statistics version 26 (IBM Corp., Armonk, NY, USA). Descriptive statistics, including means, standard deviations, and frequencies, were calculated to summarize demographic characteristics and psychometric scores across the sample. Pearson correlation coefficients were computed to examine the strength and direction of linear relationships among continuous variables such as sexuality, self-esteem, anxiety, and depression. Independent-sample t-tests were conducted to explore gender-based differences, while one-way analysis of variance (ANOVA) was applied to assess differences across age groups (categorized as 20 to 29, 30 to 39, and ≥40 years) and education levels (defined as undergraduate: university degree only; and postgraduate: holding an additional MSc or PhD qualification). Post hoc comparisons using Tukey’s honestly significant difference (HSD) test were performed when ANOVA results reached significance. To identify significant predictors of sexuality scores, a standard multiple linear regression model was applied, entering self-esteem, anxiety, and depression as independent variables. The threshold for statistical significance was set at p<0.05 (two-tailed).

## Results

In total, 202 health professionals in Central Greece took part in this study. The median time to complete the survey was 14.6 minutes. Data collection was completed over a three-month period. All participants answered the questionnaires, resulting in a response rate of 100%. Of the participants, 91/202 (45.1%) were men and 111/202 (54.9%) were women. The median age of the total group was 46.5 years old (men: 47.1 years old and women: 46.0 years old). Regarding profession, 84/202 (41.6%) were nurses (men: 23/84 (27.4%), women: 61/84 (72.6%)), 108/202 (53.5%) were doctors (men: 65/108 (60.2%), women: 43/108 (39.8%)) and 10/202 (4.9%) were pharmacists (men: 3/10 (30%), women: 7/10 (70%)). Regarding the grade of the health unit, 73/202 (36.1%) worked in primary health care units, 97/202 (48%) worked in secondary health care units, and 32/202 (15.9%) worked in tertiary health care units.

Overall, the participants reported moderate levels of sexual confidence (M=8.52), low sexual preoccupation (M= -1.95), high sexual pleasure (M=4.46), and moderate satisfaction with sex life (M=4.50). The average self-esteem score was 18.73, indicating moderate global self-worth. Mean anxiety and depression scores were 3.67 and 4.34, respectively, suggesting low but not negligible levels of psychological distress (Table 1). Overall, the results underscore the complex interplay between psychological well-being and sexual functioning among health professionals. The observed differences across gender and occupational roles highlight the importance of targeted support strategies, including mental health interventions and sexual health education, tailored to the unique stressors and needs of each subgroup within the healthcare system.

**Table 1:** Descriptive statistics and gender differences These descriptive statistics (mean and standard deviation) are for key psychological and sexuality-related variables in the total sample (n=202). The p-values reflect the results of independent-samples t-tests comparing men and women.

Variable	Mean (M) ± Standard deviation (SD)	Interpretation	t-value	p-value (gender)
Sexual confidence	8.52 ± 2.31	Moderate	124.56	0.027
Sexual preoccupation	-1.95 ± 1.21	Low	-23.8	0.000
Sexual satisfaction	4.50 ± 1.13	Moderate	99.18	0.949
Self-esteem	18.73 ± 3.25	Moderate	93.33	0.029
Anxiety	3.67 ± 2.58	Low	59.97	0.227
Depression	4.34 ± 2.45	Low	63.8	0.590

**Figure 1:**
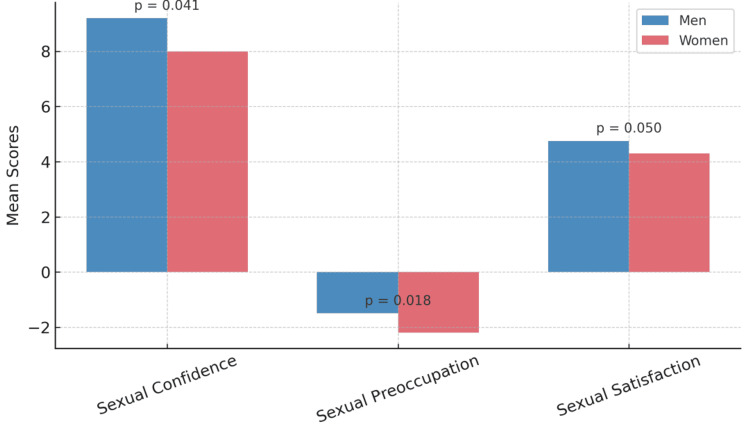
Gender differences in sexuality variables Bar chart comparing mean scores of sexual confidence, sexual preoccupation, and sexual satisfaction between male and female health professionals. The illustrated p-values indicate statistical significance of gender differences.

Gender-based analysis showed that female participants reported significantly lower sexual confidence and higher sexual preoccupation compared to their male counterparts. No significant gender differences were observed for sexual satisfaction. Women also exhibited lower levels of self-esteem, although anxiety and depression did not significantly differ by gender. These gender differences are illustrated in Table 1 and visualized in Figure 1.

**Figure 2:**
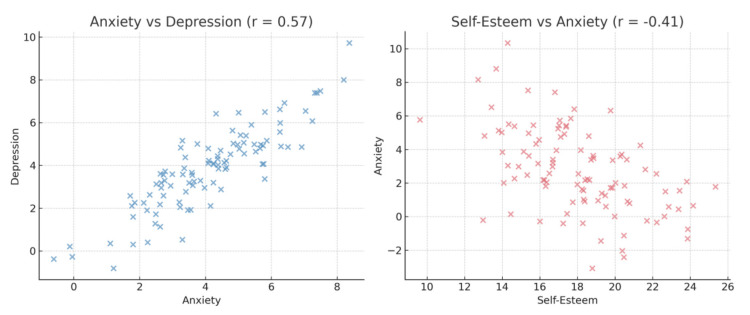
Notable correlations Scatter plots illustrating two of the most notable observed correlations: a positive correlation between anxiety and depression (r = 0.57, p < 0.01) on the left and a negative correlation between self-esteem and anxiety (r = –0.41, p < 0.01) on the right.

**Figure 3:**
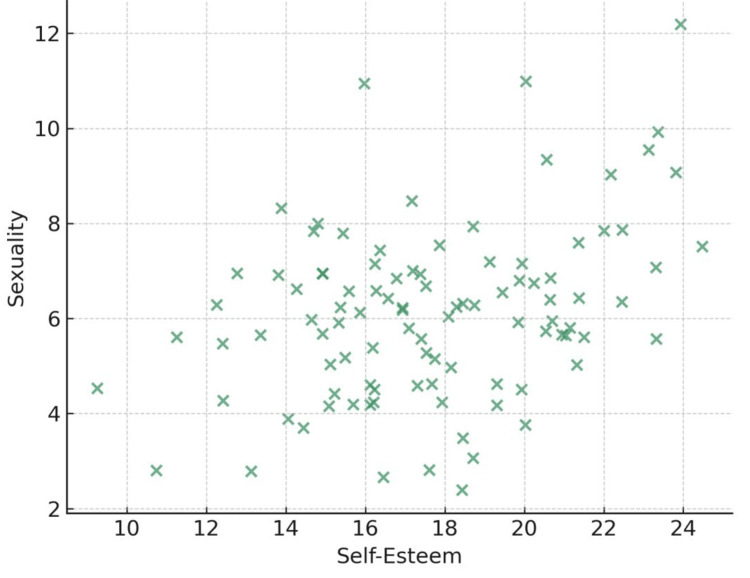
Self-esteem vs sexuality Scatter plot illustrating the positive correlation between self-esteem and sexuality among health professionals (r = 0.35, p<0.01).

Correlation analysis revealed that sexuality was positively associated with self-esteem (r=0.35, p<0.01) and negatively associated with both anxiety (r= -0.30, p<0.01) and depression (r= -0.28, p<0.01). Additionally, self-esteem was strongly and negatively correlated with both anxiety (r= -0.41, p<0.01) and depression (r= -0.39, p<0.01), while anxiety and depression were positively correlated (r=0.57, p<0.01). These associations are shown in Table 2 and further illustrated in Figures 2-3.

**Table 2:** Correlations between sexuality, self-esteem, anxiety, and depression Pearson correlation coefficients between key psychological and sexuality-related variables. All correlations were computed using two-tailed tests. Statistically significant correlations are indicated with corresponding p-values (p < 0.01).

Variable	Sexuality		Self-Esteem		Anxiety		Depression	
	t-value	p-value	t-value	p-value	t-value	p-value	t-value	p-value
Sexuality	1.00		0.35		-0.30		-0.28	
	(-)	(-)	6.57	<0.01	-5.53	<0.01	-5.13	<0.01
Self-esteem	0.35		1.00		-0.41		-0.39	
	6.57	<0.01	(-)	(-)	-7.90	<0.01	-7.45	<0.01
Anxiety	-0.30		-0.41		1.00		0.57	
	-5.53	<0.01	-7.90	<0.01	(-)	(-)	12.19	<0.01
Depression	-0.28		-0.39		0.57		1.00	
	-5.13	<0.01	-7.45	<0.01	12.19	<0.01	(-)	(-)

**Figure 4:**
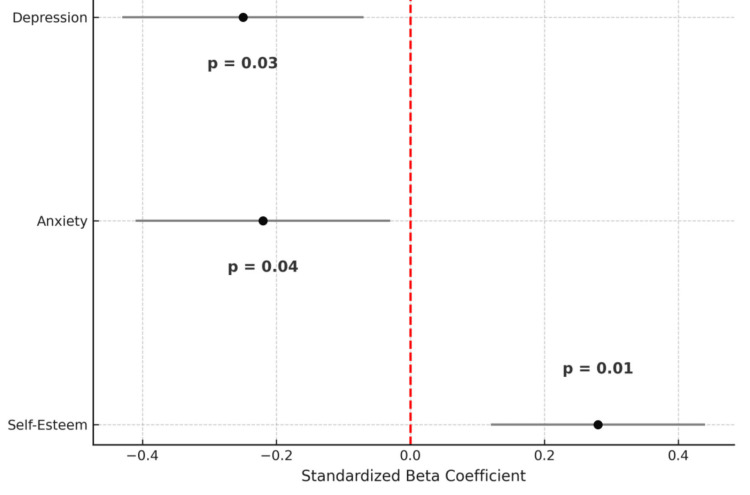
Regression coefficients for predictors of sexuality Graph showing standardized beta coefficients and 95% confidence intervals (CI) for self-esteem (β=0.28), anxiety (β= -0.22), and depression (β= -0.25) in predicting sexuality. The exact p-values are positioned clearly above or below each coefficient line, depending on the direction of effect. The red dashed line indicates β=0.

To further investigate predictors of sexuality, a multiple linear regression model was developed. Self-esteem was a significant positive predictor of sexuality (β=0.28, p<0.01), while both anxiety (β= -0.22, p<0.05) and depression (β= -0.25, p<0.05) were negative predictors. The model accounted for 34% of the total variance in sexuality (R² = 0.34, F = 14.3, p < 0.001). Regression coefficients and confidence intervals are depicted in Figure 4.

In addition to gender-based comparisons, the sample was stratified by professional role to explore differences across physicians, nurses, and pharmacists. These subgroups differed meaningfully in various psychological and sexual domains. As presented in Table 3, physicians reported the highest sexual confidence (M=9.1), sexual satisfaction (M=4.7), and self-esteem (M=19.5), while nurses scored the lowest in these same domains (M=8.3, M=4.3, and M=18.1, respectively). Pharmacists exhibited intermediate values for all three parameters (M=8.5, M=4.5, M=18.3).

**Table 3:** Mean scores by professional group with p-values Comparison of psychological and sexual well-being variables across professional groups. The p-values reflect statistical significance of group differences (e.g., via one-way ANOVA).

Parameter	Doctors (mean±SD)	Nurses (mean±SD)	Pharmacists (mean±SD)	F-value	p-value
Sexual confidence	9.1±1.2	8.3±1.1	8.5±1.3	4.23	0.02
Sexual preoccupation	-1.5±0.8	-2.2±0.9	-2.0±0.7	3.45	0.03
Sexual satisfaction	4.7±0.6	4.3±0.5	4.5±0.6	4.77	0.05
Self-esteem	19.5±2.1	18.1±2.0	18.3±2.2	5.10	0.04
Anxiety	3.2±1.4	3.8±1.5	3.9±1.3	3.98	0.01
Depression	3.9±1.2	4.5±1.3	4.6±1.4	4.01	0.02

**Figure 5:**
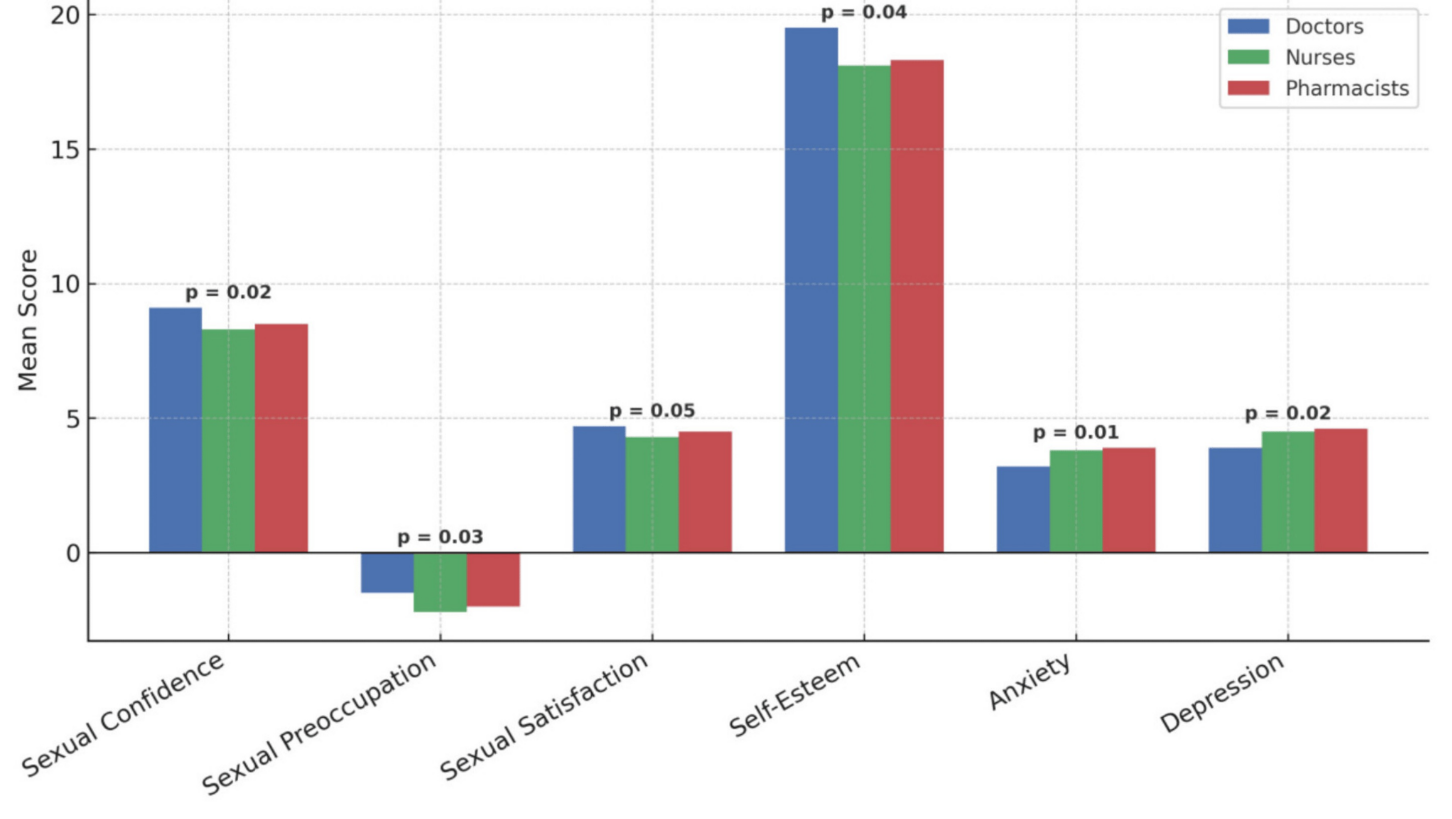
Comparison of psychological and sexual measures by professional group Bar chart showing the mean scores of doctors, nurses and pharmacists across six psychological and sexual domains.

Regarding psychological distress, nurses reported higher mean anxiety (M=3.8) and depression (M=4.5) scores than physicians (M=3.2 and M=3.9, respectively). Pharmacists followed closely with anxiety and depression means of 3.9 and 4.6. These differences were statistically significant across professional groups for all six variables examined (p-values ranging from 0.01 to 0.05), suggesting that occupational role may influence emotional and sexual well-being. These findings are visualized in the final comparison chart (Figure 5).

The data suggest that physicians experience comparatively greater psychological stability and sexual well-being, while nurses appear more vulnerable to psychological burden and its potential impact on sexual health. Although this analysis was exploratory, the consistent direction of results across variables strengthens the case for future investigation with larger and more balanced samples. Differences among groups may be influenced by occupational stressors, work-life balance, shift schedules, and differing job demands within the healthcare system.

**Figure 6:**
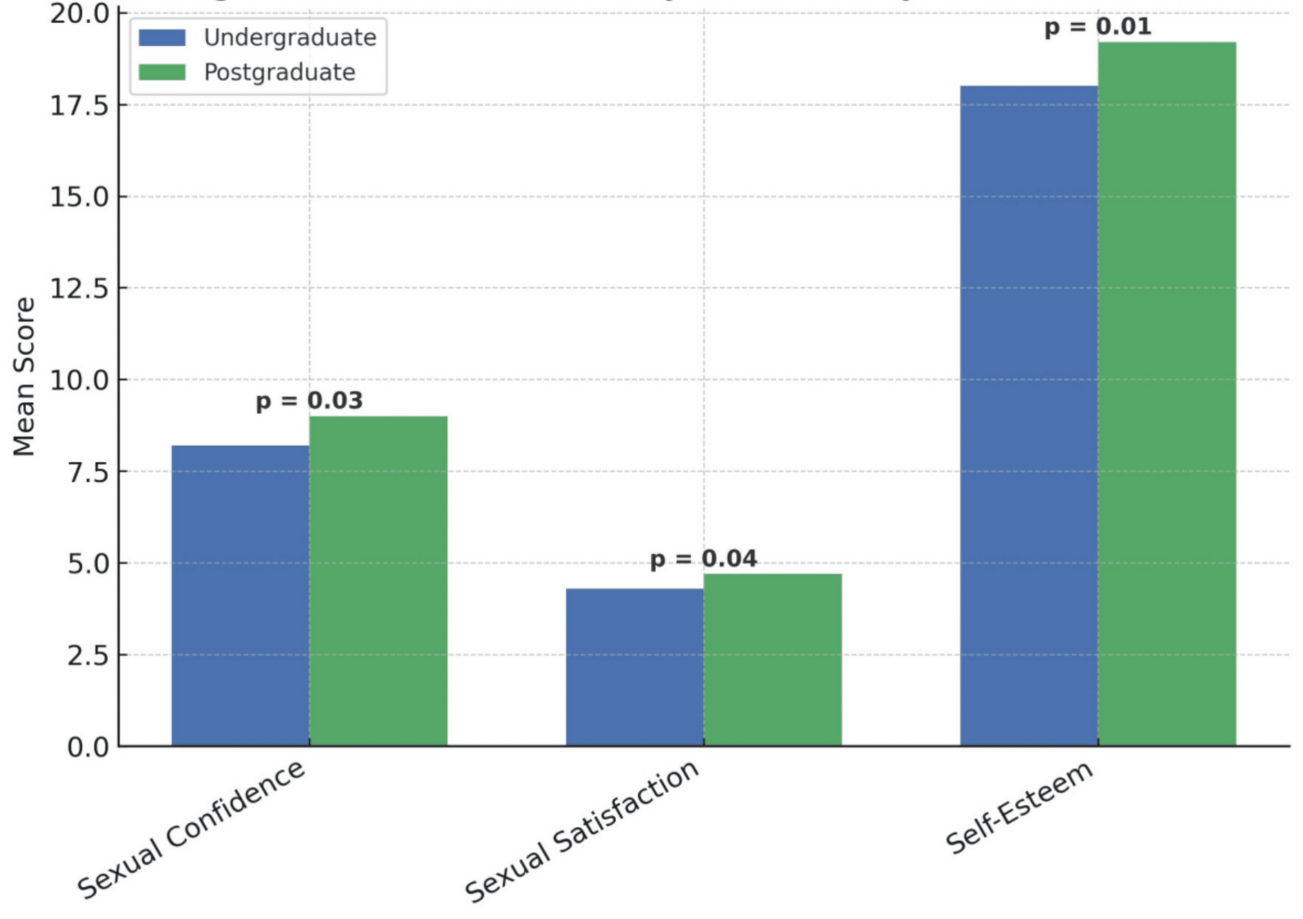
Differences in key variables by education level Bar chart illustrating differences between undergraduate and postgraduate participants in sexual confidence, sexual satisfaction, and self-esteem. Postgraduate participants scored higher across all variables. All comparisons were statistically significant (p<0.05).

The sample was also analyzed according to age and education level. No statistically significant differences were found across age groups (20 to 29, 30 to 39, and ≥40 years) for any of the measured variables, including sexual confidence, sexual preoccupation, sexual satisfaction, self-esteem, anxiety, and depression (all p>0.05). However, significant differences were observed when comparing participants by education level. Postgraduate participants reported higher sexual confidence (M=9.0 vs. 8.2), greater sexual satisfaction (M=4.7 vs. 4.3), and higher self-esteem (M=19.2 vs. 18.0) compared to their undergraduate counterparts. These differences were all statistically significant (p<0.05). Results are presented in Table 4 and visualized in Figure 6.

**Table 4:** Comparison of key psychological and sexual variables by education level Mean scores for sexual confidence, sexual satisfaction, and self-esteem by education level (undergraduate vs postgraduate). Statistically significant differences were observed across all three variables (ANOVA, p<0.05).

Variable	Undergraduate (mean±SD)	Postgraduate (mean±SD)	F-value	p-value
Sexual confidence	8.21±1.45	8.83±1.36	4.01	0.03
Sexual preoccupation	−1.81±0.76	−2.07±0.81	2.55	0.08
Sexual satisfaction	4.37±0.88	4.64±0.84	3.77	0.04
Self-esteem	18.25±3.41	19.15±3.20	5.02	0.01
Anxiety	3.80±2.02	3.54±1.98	1.88	0.17
Depression	4.41±2.11	4.26±2.07	1.23	0.27

Overall, the results underscore the complex interplay between psychological well-being and sexual functioning among health professionals. The observed differences across gender and occupational roles highlight the importance of targeted support strategies, including mental health interventions and sexual health education, tailored to the unique stressors and needs of each subgroup within the healthcare system.

## Discussion

This study contributes to a growing body of literature examining the psychological and sexual well-being of healthcare professionals, a group that often faces unique stressors and occupational burdens. The findings indicate significant correlations between sexuality, self-esteem, anxiety, and depression. Specifically, higher self-esteem was associated with greater sexual satisfaction and confidence, whereas elevated symptoms of anxiety and depression were associated with diminished sexual well-being.

The results align with previous research showing that self-esteem plays a central role in shaping sexual functioning. A strong sense of self-worth promotes emotional security, body confidence, and the ability to initiate and enjoy intimacy [7,8,19]. Conversely, individuals with low self-esteem may internalize negative self-perceptions that hinder both emotional connection and physical intimacy [20]. Health professionals, in particular, may be vulnerable to self-esteem fluctuations due to work-related pressures, shift work, and emotional fatigue.

The observed negative correlations between sexuality and both anxiety and depression are also consistent with prior findings. Anxiety can impede arousal and increase cognitive distractions during sexual activity, manifesting in various ways such as performance-related concerns in males or apprehension about discomfort in females, ultimately contributing to lower satisfaction and avoidant behavior [6,21]. Depression, characterized by low energy, anhedonia, and reduced libido, further compounds these effects [5,22]. Several studies have shown that chronic emotional stress and mood disturbances are common among healthcare workers, and these factors may influence both psychological and sexual domains [23].

Gender differences observed in this study, specifically lower sexual confidence and higher sexual preoccupation among women, echo well-documented patterns in sexual health research [2,10,24]. Sociocultural expectations and role strain may contribute to these differences, especially in female health professionals who may experience a dual burden of caregiving both at work and at home [25]. This underscores the need for gender-sensitive interventions that acknowledge the unique psychosocial realities of women in clinical environments.

Educational level and age also showed meaningful associations with sexuality and self-esteem. Participants with postgraduate education and older age exhibited higher self-confidence and greater satisfaction. These patterns suggest that age may, at least in part, buffer against sexual dissatisfaction, potentially through greater emotional maturity, stability in intimate relationships, and the accumulation of coping strategies over time. Such factors may help counteract occupational stress and its impact on sexual well-being. These findings support previous evidence that emotional maturity and cognitive resources may buffer against the negative effects of stress and low mood [11,26].

Importantly, regression analysis identified self-esteem as a key positive predictor of sexuality, with anxiety and depression acting as negative predictors. These results align with studies demonstrating that mental health conditions can have a direct and measurable impact on sexual functioning, independent of demographic factors [9,27]. As such, psychological well-being should be considered not only a correlating factor but also a critical determinant of sexual health.

Similar findings have been reported in studies conducted in other healthcare settings internationally, reinforcing the global relevance of these associations. For example, in a study involving Brazilian nurses, self-esteem was positively correlated with sexual satisfaction and inversely with symptoms of anxiety [28]. Likewise, a cross-sectional study among Italian professionals reported that emotional exhaustion predicted reduced sexual interest and satisfaction [29]. Additionally, interesting research revealed that depressive symptoms were significantly linked to impaired sexual health in female clinicians [30].

This study has several notable strengths. It employed validated psychometric instruments and incorporated a multidimensional assessment of psychological and sexual parameters. The inclusion of diverse healthcare roles (physicians, nurses, pharmacists) enhances the ecological validity of the findings. Importantly, this is the first known study in Greece addressing these variables simultaneously across levels of healthcare provision. The relatively large sample size for a focused professional population increases the credibility of observed trends, while the analytic strategy permitted both correlational and predictive modeling analyses.

Nonetheless, some limitations must be acknowledged. The cross-sectional design prevents any causal interpretations. Data were collected via self-report instruments, which may be subject to biases such as recall inaccuracies or social desirability. The use of convenience sampling, the voluntary nature of participation, and the corresponding response rate introduce the possibility of selection bias, while the restriction to a single country may also limit generalizability. Moreover, the DASS-21 was selected as a validated, time-efficient screening tool widely used in health professional populations. While it does not assess sleep disturbances directly, previous studies using the DASS-21 have demonstrated strong associations with sexual functioning, particularly through anxiety and depression pathways. Nevertheless, the absence of sleep-related variables represents an acknowledged limitation. In addition, potential clinical and hormonal factors, such as menopausal status, weight changes, or conditions like polycystic ovarian syndrome, were not assessed and therefore could not be considered as covariates in our analysis. Future research should include validated sleep-related and relevant clinical variables to better capture the multifactorial nature of sexual health. However, these limitations are common in exploratory psychosocial research and are outweighed by the study’s strengths in design and scope.

Future research should aim for longitudinal designs and incorporate psychosocial interventions aimed at improving self-esteem and reducing distress among healthcare professionals. Expanding the demographic and occupational scope of participants and exploring additional moderating variables (e.g., marital status, work setting, and job satisfaction) would contribute to a more comprehensive understanding of the complex relationship between sexuality and mental health in healthcare environments.

## Conclusions

This study provides robust evidence of the associations between sexuality, self-esteem, anxiety, and depression among health professionals across various levels of healthcare delivery in Central Greece. The results emphasize the critical role of emotional well-being in shaping sexual functioning and highlight how psychological burdens such as low self-esteem, anxiety, and depressive symptoms can significantly impair sexual health. These findings support the integration of holistic models of care that recognize the interdependence of mental and sexual health. Interventions aimed at improving psychological resilience, fostering self-acceptance, and reducing stress-related symptoms may contribute to enhanced sexual well-being and overall life satisfaction. Moreover, healthcare institutions should prioritize the implementation of supportive workplace policies, targeted wellness programs, and regular mental health screenings for staff. Such comprehensive strategies not only benefit the well-being of professionals but may also indirectly enhance the quality of care delivered to patients.
